# Comparison of metagenomic analysis of fecal and gastrointestinal tract samples for identifying beneficial gut microorganisms

**DOI:** 10.3389/fmicb.2025.1533580

**Published:** 2025-03-26

**Authors:** Ji-Seon Ahn, Eui-Jeong Han, Hea-Jong Chung

**Affiliations:** ^1^Honam Regional Center, Korea Basic Science Institute, Gwangju, Republic of Korea; ^2^College of Pharmacy, Chung-Ang University, Seoul, Republic of Korea; ^3^Department of Bio-Analysis Science, University of Science & Technology, Daejeon, Republic of Korea

**Keywords:** metagenomic analysis, differential analysis, motor function, cognitive function, emotional function

## Abstract

**Introduction:**

Previous research on the gut microbiome has primarily focused on fecal microbiota, raising concerns about whether fecal samples accurately represent the entire intestinal microbiota. Studies have shown that microbial communities across the gastrointestinal (GI) tract are more diverse than those in feces, suggesting that microbial composition may vary depending on the sampling method. Additionally, analyzing the broader diversity of microbial communities in the GI tract may enhance the identification of potentially beneficial microbiota.

**Methods:**

In this study, we compare gut microbiome datasets obtained from fecal samples and GI samples (collected by pooling luminal contents and mucosal scrapings from the stomach to the end of the colon) of 6-month-old mice using 16S rRNA sequencing. We further investigate the associations between gut microbiota and motor, cognitive, and emotional functions in mice, examining differences between the two sample types. To assess these variations, we apply DESeq2 analysis to identify microbial species enriched in high-functioning groups and evaluate how their selection may differ depending on the sampling approach.

**Results:**

Our findings reveal notable differences in microbial composition between fecal and GI samples, suggesting that sampling methods may influence the identification of beneficial bacteria.

**Discussion:**

These results highlight the importance of selecting an appropriate sampling approach in microbiome research to ensure a comprehensive understanding of gut microbiota-host interactions.

## 1 Introduction

The gut microbiome is a complex microbial community comprising 100 trillion microorganisms present in the digestive tract (Chen et al., 2013). Because the gut microbiome influences host phenotype, including host health and disease, there has been a large field of research on the gut microbiome and its role in host phenotype and disease (Chen et al., 2013; Chung et al., 2018; Liu et al., 2018; Lkhagva et al., 2021; Nguyen et al., 2017; Nguyen et al., 2019).

Previous studies have clearly shown that host locomotion and muscle strength can be regulated by gut microbiota (Ahn et al., 2023a; Ahn et al., 2024). Additionally, there are research results on the relation-ship between intestinal microorganisms and cognitive and emotional functions (Liang et al., 2022; Shoubridge et al., 2022). Given the significant impact of gut microbiota on host traits, the concept of “microbiability” has been proposed to describe the extent to which gut microbes influence phenotypic traits of the host (He et al., 2022). However, despite research into these associations, few studies have identified the exact gut microbial species responsible for the host phenotype.

Gut microbiota research is typically conducted through fecal microbiome analysis, but it is important to note that the fecal microbiome does not always represent the entire gut microbiome. This is because the environment, such as pH and moisture, varies depending on the location of the gastrointestinal tract within the entire intestine (Ahn et al., 2023b). In other words, it is important to recognize that in gut microbiome research, the method of sample collection can affect the interpretation of results.

In this study, we aim to compare microbial diversity and composition between fecal and gastrointestinal samples from 6-month-old mice, with a particular focus on understanding how these differences may impact the correlation between gut microbiota and host behaviors, including motor, cognitive, and emotional functions. To clarify the distinction between sample types, we define gastrointestinal (GI) samples as those collected by pooling luminal contents and mucosal scrapings from the stomach to the end of the large intestine after sacrifice. Through 16S rRNA sequencing of both fecal and GI samples, we seek to identify microbial species associated with specific host phenotypes and assess how differences in sampling methods may influence microbiome analysis outcomes.

## 2 Materials and methods

### 2.1 Study design and animal experiment

Thirty-five C57BL/6J mice (5-month-old, 14 females and 21 males) were obtained from the Animal Facility of Aging Science in Korea Basic Science Institute (Gwangju, Republic of Korea). The mice were individually housed in a specific-pathogen-free (SPF) facility with *ad libitum* access to sterilized food and water. The housing environment was maintained at 22 ± 1°C and 40–50% humidity with a 12 h light/dark cycle. Microbiological monitoring in a specific-pathogen-free (SPF) facility was performed using Laser scanning confocal microscope (Leica TCS SP5 AOBS/Tandem, KJ302) at the Honam Regional Center of Korea Basic Science Institute (KBSI). The entire experiment was conducted within 1 week after the stabilization period. Following body weight measurement and blood collection, planned behavioral analysis experiments were conducted. All tests were performed during the light phase (09:00–18:00) to ensure consistency. After all behavioral analyses were completed, excreted feces (Feces) and gastrointestinal tract (GI) samples were collected for microbiome analysis. Freshly excreted feces were obtained from each mouse prior to sacrifice, representing the microbiota expelled from the gut. To collect GI samples, mice underwent a 24-h fasting period before sacrifice to minimize residual food contents in the gut. After sacrifice, the entire gastrointestinal tract, from the stomach to the end of the large intestine, was carefully dissected. The luminal contents and mucosal surfaces were collected by scraping the inner lining of the digestive tract, ensuring that the GI samples contained both luminal microbiota and potentially mucosa-associated microbiota.

### 2.2 Analyses of biochemical parameters

The serum levels of total cholesterol (TCHO), triglyceride (TG), high-density lipoprotein-cholesterol (HDL-CHO) and blood glucose levels were determined using commercial assay kits (Asan Pharmaceutical, Seoul, Republic of Korea) following the manufacturer’s instructions, and the low-density lipoprotein-cholesterol (LDL-CHO) levels were calculated using Friedewald’s equation, as previously described (Baek et al., 2014; Chung et al., 2016).

### 2.3 Mouse behavior tests

This protocol describes four behavioral tasks for mice to assess motor function, cognitive function, and emotional function. To investigate the relationship between microbial diversity and host behavior, the rotarod test, wire suspension test, Y-maze spontaneous alternation test, and tail suspension test were conducted. Mice were categorized into high, medium, and low groups based on performance in behavioral tests.

#### 2.3.1 Rotarod test

To assess motor coordination, balance, and muscle strength, we employed a rotarod apparatus (B. S. Technolab Inc., Seoul, South Korea). The testing protocol followed an established methods in which the rotarod was accelerated from rest to 30 rpm over 5 min, as previously described (Ahn et al., 2024). Each mouse underwent three trials, each lasting up to 5 min, and the time spent on the rod (latency to fall) was used for subsequent analysis. Mice with recording times of less than 10 s, even in one experiment, were excluded.

#### 2.3.2 Wire suspension test

To assess forelimb strength, we used a wire suspension apparatus consisting of two vertical supports (60 cm apart) connected by a stainless steel wire (50 cm in length, 2 mm in diameter). Following an established method (Ahn et al., 2023a), each mouse was placed on the wire using only its forepaws, and the latency to fall was measured. Each mouse underwent three trials, and the average latency was used for analysis. Mice with recording times of less than 10 s in any trial were excluded. To classify mice based on motor function, we combined the ranks from both the rotarod and wire suspension tests. Mice were categorized into high, medium, and low motor function groups using a percentile-based ranking method, ensuring consistency with the Results section.

#### 2.3.3 Y-maze spontaneous alternation test

The Y-maze spontaneous alternation test assessed the mice’s short-term working memory. The testing protocol followed an established method, as described previously (Kraeuter et al., 2019). Each mouse was placed in arm A and allowed to freely explore the maze for 8 min. We tracked the sequence of arms each mouse entered, with a complete entry requiring all four paws inside an arm. The test measured “spontaneous alternation,” where mice entered each of the three arms consecutively in a different order, without revisiting any arm within a sequence of three entries. The percentage alternations were calculated as the number of actual alternations divided by the maximum number of alternations (the total number of arm entries −2). Mice were categorized into high, middle, and low groups based on their performance in the spontaneous alternation Y-maze test, which measures cognitive function. Mice with a total number of entries less than 30 were excluded. Mice with a total number of entries less than 30 were excluded.

### 2.3.4 Tail suspension test

Depression-like behavior was examined using the tail suspension test described previously (Ueno et al., 2022). The testing apparatus consisted of white acrylic walls (20 × 30 × 60 cm) and one open side for observation. Each mouse was suspended by its tail 60 cm above the chamber floor using adhesive tape placed near the tip of its tail (less than 1 cm). The mouse’s behavior was observed by an observer for 6 min and the time of immobility was recorded. Mice were classified into high (more movement), medium, and low (less movement, depressed) groups based on immobility time in the tail suspension test, a measure of emotional despair.

### 2.4 DNA extraction and 16S rRNA gene sequencing

The DNA from each individual fecal and gastrointestinal (GI) sample was extracted using the phenol-chloroform-isoamyl alcohol extraction method, following the same procedure as previously described (Ahn et al., 2023a; Ahn et al., 2024; Zhai et al., 2022). To ensure DNA quality, both concentration and purity were measured using a BioSpec-nano spectrophotometer (Shimadzu Biotech, Japan), and DNA integrity was confirmed using 1% (w/v) agarose gel electrophoresis. The extracted DNA was sent to a specialized sequencing facility (Ebiogen, Inc., Republic of Korea) for Next-Generation Sequencing (NGS) using the Illumina 16S Metagenomic Sequencing Library protocol. This involved amplifying the V3–V4 region of the 16S rRNA gene using designed primers, as previously described (Ahn et al., 2023a; Ahn et al., 2024; Zhai et al., 2022). After amplification, additional steps were performed to incorporate a multiplexing index and Illumina sequencing adapters. Each sample underwent individual normalization and pooling once before sequencing, utilizing PicoGreen. Library size confirmation was performed using the Agilent TapeStation DNA ScreenTape D1000 system (Agilent Technologies, Santa Clara, CA, United States). The final pooled libraries underwent sequencing (2 × 300 paired-end reads) using the Illumina MiSeq platform (Illumina, San Diego, CA, United States). Amplicon error correction was performed by modeling from merged fastq files using DADA2 (Ver. 1.10.1). This process included filtering out noise sequences, correcting errors in marginal sequences, removing chimeric sequences and singletons, and de-replicating sequences (Callahan et al., 2016a).

### 2.5 Microbial diversity and abundance analyses

All data and statistical analyses were conducted as previously described (Ahn et al., 2023a; Ahn et al., 2024; Zhai et al., 2022). Briefly, bacterial species were classified using Q2-Feature Classifier, a Naive Bayes classifier trained on the SILVA reference (V3–V4 region) database.^1^ Following parameter configuration using the Denoise single function, the dataset was classified. Diversity calculations and statistical tests were conducted utilizing the q2 diversity option, specifically focusing on “sampling depth.” A minimum sequencing quality score threshold of 20 and a rarefaction depth of 11,510 were applied. Subsequently, upon validating the sequencing results’ quality, the “table.qzv” file was filtered using thresholds in QIIME 2. The metagenomic data OTU and taxonomic classification tables were imported into R version with the phyloseq (1.28.0) and MetagenomeSeq (version 1.16.0) packages. Following established guidelines, we imported metadata, OTU, and taxonomic classification tables into phyloseq, where the data was processed (Callahan et al., 2016b). To normalized the data specifically for metagenomic analysis, we converted phyloseq objects to MetagenomeSeq objects and applied Cumulative Sum Scaling (CSS) from the Bioconductor package metagenomeSeq (version 1.16.0). Finally, the normalized data was converted back to phyloseq class objects for further analysis and visualization in R (version 3.6.1). The α-diversity, β-diversity analyses and relative abundance evaluation were conducted as previously described (Ahn et al., 2023a; Ahn et al., 2024; Zhai et al., 2022). The α-diversity was statistically analyzed using the Kruskal–Wallis rank sum test. The β-diversity was computed using Bray–Curtis dissimilarity on log-transformed OTU data. Non-metric multidimensional scaling (NMDS) was employed using the “MetaMDS” function from the “vegan” package to assess relationships between samples by reducing dimensionality while retaining information on sample relationships. For abundance calculations, the normalized OTU data were utilized, and taxa were grouped at the phylum level for plotting. To enhance clarity in visualizing abundance data, classification groups with a relative abundance of less than 0.5% were aggregated as “Others.”

### 2.6 Differential abundance analysis

To identify differences in bacterial populations between the two groups following each behavioral test, differential abundance analysis was executed following established procedures (Ahn et al., 2023a; Ahn et al., 2024). Utilizing DESeq2 (version 1.24.0), we analyzed the differences in the types and amounts of bacteria found in different groups. This analysis focused on Feces and GI samples collected after each behavioral test (Love et al., 2014; Paulson et al., 2013). Taxonomic species present in less than 1% of the samples were disregarded in the DESeq2 analysis.

### 2.7 Calculation of microbiability

Microbiability was calculated to estimate the proportion of phenotypic variance explained by the abundance of specific microbial species (He et al., 2022). For each selected species, a linear model (lm) was fitted using the species abundance as the fixed effect and the host phenotype as the dependent variable. The total phenotypic variance was computed, and the contribution of each microbial species was determined based on the model’s *R*^2^ value. Microbiability was then obtained by dividing the species-specific variance by the total phenotypic variance. To assess the statistical significance of the association between microbial species and host phenotypes, *p*-values were extracted from the regression model coefficients. The *p*-values corresponding to the microbial abundance variable were reported to evaluate whether the observed microbiability was statistically significant. All calculations were performed in R (version 4.3.3) using standard statistical packages.

### 2.8 Statistical analysis

All statistical analyses are reported as the mean ± S.E.M., and the differences in the relative abundance of bacterial populations containing feces and GI were analyzed using the Mann–Whitney sum rank tests in R software. Group comparisons in the NMDS plots were statistically analyzed using the PERMANOVA test, while most other group comparisons were performed using Welch’s *t*-test. The *p*-value less than 0.05 was considered significant.

## 3 Results

### 3.1 Microbial diversity comparison: fecal vs. gastrointestinal samples

**Figure 1:**
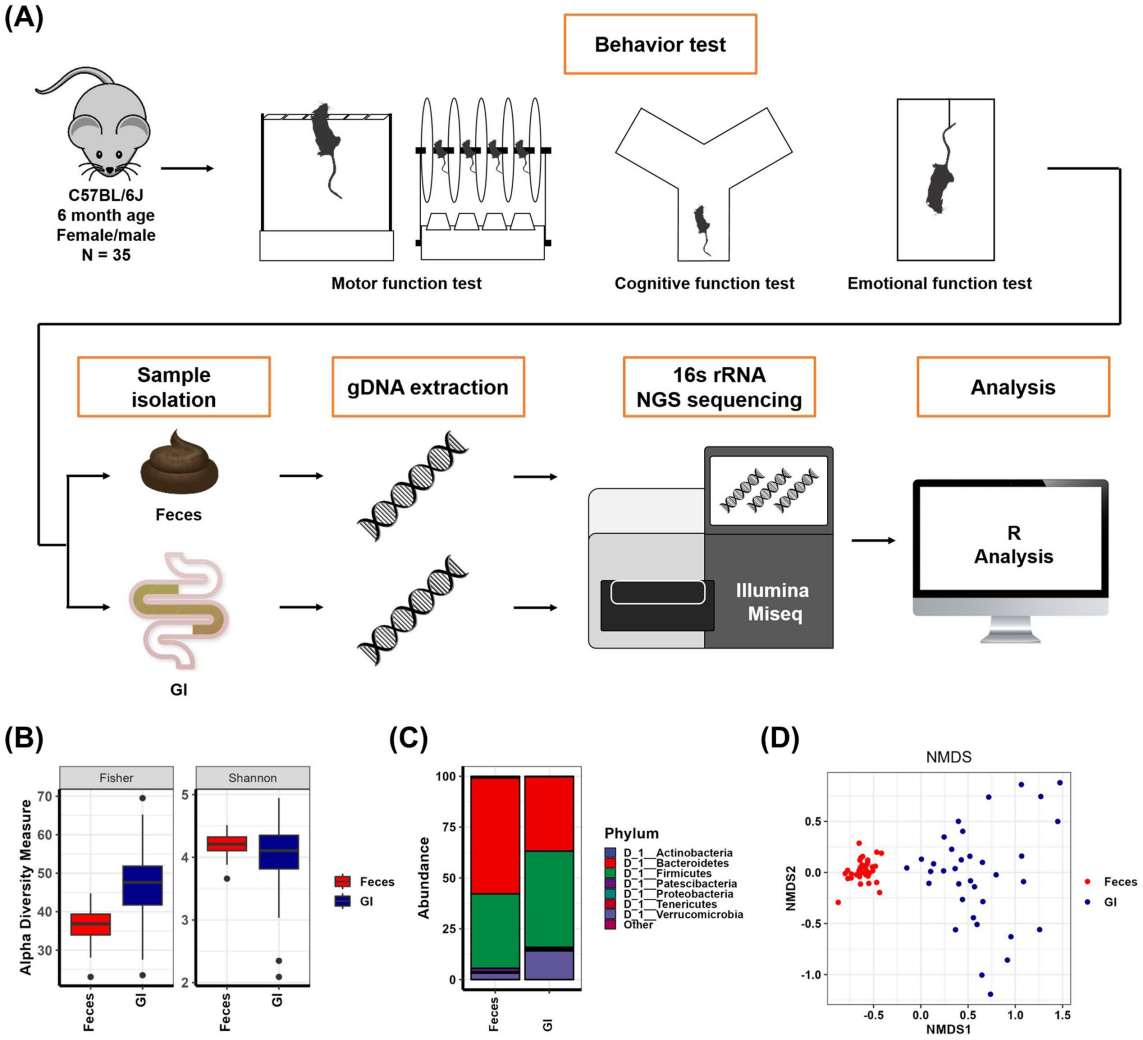
The gut microbiota obtained from feces and the gut microbiota obtained from the whole gastrointestinal tract are different. **(A)** Schematic diagram of mice behavior tests and metagenomic analysis. **(B)** Alpha-diversity indices of Feces samples and GI samples. The mean and *p*-value are presented in Supplementary Table S1. **(C)** Abundance (%) in Feces samples and GI samples at the phylum level. The mean and *p*-value are presented in Supplementary Table S2. **(D)** Nonmetric multidimensional scaling plot of Feces samples and GI samples. The results and *p*-values of the PERMANOVA test using Adonis analysis are in Supplementary Table S3.

In order to compare fecal microbiomes and gastrointestinal microorganisms and discover useful intestinal microbiomes, 16S rRNA sequencing of feces (Feces) and gastrointestinal tract (GI) was performed after behavioral analysis experiments on 35 6-month-old mice (Figure 1A). As a result of comparing the alpha diversity of Feces samples and GI samples, it was observed that the richness factors Fisher (*p* < 0.001) and ACE (*p* < 0.001) were larger in GI samples, and the evenness factors Shannon (*p* < 0.05), Simpson (*p* < 0.001), InvSimpson (*p* < 0.001), and Evenness (*p* < 0.001) were slightly smaller in GI samples than in Feces samples (Figure 1B and Supplementary Table S1). In more detail, as a result of checking the microbial composition of the Feces samples and the GI samples, it was confirmed that Actinobacteria (*p* < 0.001), Bacteroidetes (*p* < 0.001), and Patescibacteria (*p* < 0.001) were reduced, and Firmicutes (*p* < 0.01), Proteobacteria (*p* < 0.01) and Verrucomicrobia (*p* < 0.001) were significantly increased in the GI samples compared to the Feces samples (Figure 1C and Supplementary Table S2). In addition, through the NMDS plot drawn as a result of the analysis of the Feces samples and GI samples, it was seen that the microbial composition between the Feces samples and GI samples was clearly different (*p* = 0.001), and that there were clear differences between each individual in GI samples compared to Feces samples (Figure 1D and Supplementary Table S3). Through this, it is assumed that it will be possible to clearly distinguish differences in intestinal microorganisms according to phenotype in GI samples rather than Feces samples.

### 3.2 Behavioral phenotype and microbial composition

To investigate the relationship between gut microbiota obtained from two sampling methods and mouse phenotypes, we evaluated motor, cognitive, and emotional functions (Supplementary Table S4). Motor function was assessed using the rotarod and wire suspension tests. Based on performance records, mice were categorized into three groups: the high group (*n* = 9; rotarod record 30.81 ± 4.52 s, wire suspension record 79.7 ± 9.9 s), the medium group (*n* = 10; rotarod record 19 ± 3.12 s, wire suspension record 44.03 ± 3.96 s), and the low group (*n* = 9; rotarod record 15.78 ± 2.04 s, wire suspension record 29 ± 3.52 s). Body weight and biochemical analyses among these groups revealed that the high group had a lower body weight than the other groups. However, blood glucose, total cholesterol (TCHO), high-density lipoprotein cholesterol (HDL-CHO), low-density lipoprotein cholesterol (LDL-CHO), and triglyceride (TG) levels in the blood remained within the normal range, with no significant differences among the groups.

Cognitive function was evaluated using the Y-maze test, and groups were classified based on the calculated percentage alternation values. Mice were divided into the high group (*n* = 10; % alternation 68.67 ± 1.31%), the medium group (*n* = 11; % alternation 59.79 ± 0.83%), and the low group (*n* = 10; % alternation 50.68 ± 0.97%). Body weight and biochemical parameters among these groups showed no significant differences, and all values remained within the normal range.

Emotional function was assessed using the tail suspension test, and mice were grouped based on their immobility time. The high group (*n* = 13) had an immobility time of 115.31 ± 6.47 s, the medium group (*n* = 10) recorded 166.03 ± 4.28 s, and the low group (*n* = 12) exhibited 197.58 ± 2.95 s. Significant differences were observed among these groups. Additionally, body weight and biochemical analyses revealed that the high group had lower body weight, blood glucose, TCHO, HDL-CHO, LDL-CHO, and TG levels compared to the other groups.

#### 3.2.1 Motor function

**Figure 2:**
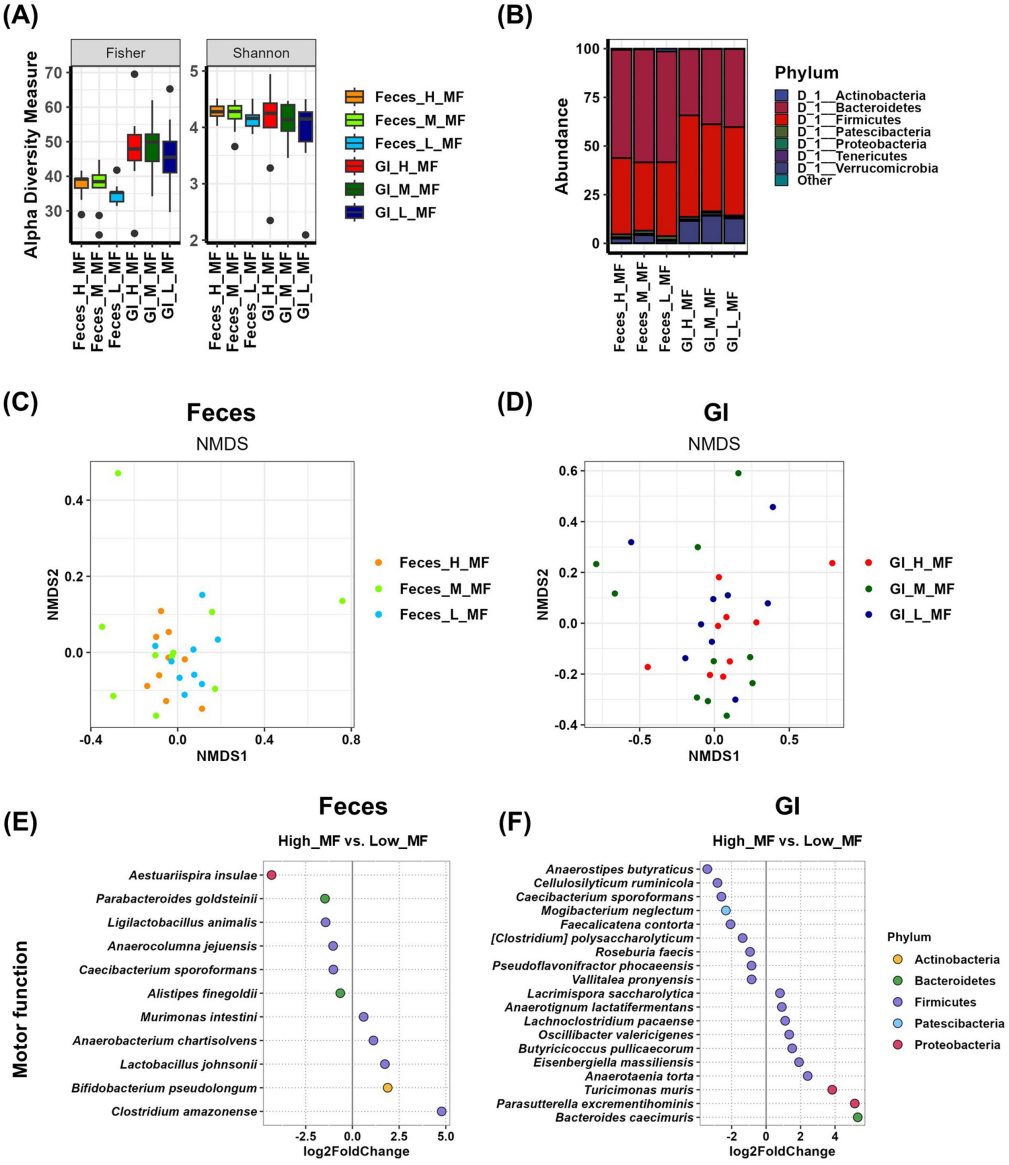
Analysis of the relationship between motor function and gut microbiota using Feces and GI tract samples. **(A)** The α-diversity indices of the Feces and GI tract microbiome measured using the Fisher and Shannon method in groups according to motor function records are shown in box plots. The mean and *p*-value for each group are presented in Supplementary Table S1. **(B)** Comparison of phylogenetic compositions in groups according to motor function records are shown in box plots of the Feces and GI tract microbiome at the phylum level. The mean and *p*-value for each group are presented in Supplementary Table S2. **(C,D)** β-diversity plot of the Feces **(C)** and GI tract **(D)** microbial communities measured using non-metric multidimensional scaling plots in the motor function-related group. The results and *p*-values of the PERMANOVA test using Adonis analysis are in Supplementary Table S3. **(E,F)** Log2-fold change in abundance of the most abundant species in the Feces **(E)** and GI tract **(F)** microbial communities of the high and low motor function groups as analyzed by DESeq2 differential abundance analysis. Each dot represents a species comparison between the two groups. The results and *p*-values of the DESeq2 test are in Supplementary Table S5. H, high group; M, medium group; L, low group; MF, motor function.

We analyzed the relationship between gut microbial communities and motor function using Feces samples and GI samples. Alpha diversity analysis showed that GI samples had higher overall richness than Feces samples, as confirmed by the Fisher index. However, no significant differences were observed in Fisher and Shannon indices across motor function groups in either Feces samples or GI samples (Figure 2A and Supplementary Table S1). In contrast, in fecal samples, evenness factors such as the Simpson and InvSimpson indices showed significant differences between the high and low motor function groups (Supplementary Table S1). Taxonomic composition analysis of Feces samples revealed that Bacteroidetes was the predominant phylum in all motor function groups (55.683% in the high group, 58.009% in the medium group, and 56.921% in the low group). Although not statistically significant, its abundance was lower in the high group compared to the other groups. Additionally, Proteobacteria abundance was slightly higher in the high group (0.444% in the high group, 0.226% in the medium group, and 0.275% in the low group), while Patescibacteria was more abundant in the low group (1.554% in the high group, 1.575% in the medium group, and 1.957% in the low group), though these differences were not statistically significant (Figure 2B and Supplementary Table S2). In GI samples, Firmicutes was the most abundant phylum (52.169% in the high group, 44.748% in the medium group, and 45.4% in the low group), with a relatively higher abundance in the high group, though not statistically significant. Conversely, Bacteroidetes abundance tended to increase with decreasing motor function (33.99% in the high group, 38.616% in the medium group, and 40.054% in the low group), but this difference was also not significant (Figure 2B and Supplementary Table S2). Beta-diversity analysis was conducted using NMDS plots, and statistical significance was assessed using the PERMANOVA test (Figures 2C,D and Supplementary Table S3). The results showed that the *p*-value for fecal samples was 0.244, while the *p*-value for GI samples was 0.406. Although neither sample exhibited statistically significant differences among motor function groups, the comparison of *p*-values suggests that fecal samples demonstrated a more systematic difference between groups than GI samples. To identify microbial species that were differentially abundant in the high and low motor function groups, we performed differential analysis using the DESeq2 program (Figures 2E,F and Supplementary Table S5). In Feces samples, six species were enriched in the high group, with *Parabacteroides goldsteinii* (*p* = 0.0053) showing a statistically significant difference. In contrast, five species were enriched in the low group (Figure 2E and Supplementary Table S5). In GI samples, nine species were enriched in the high group. Among these, *Cellulosilyticum ruminicola* (*p* = 0.0095) and *Pseudoflavonifractor phocaeensis* (*p* = 0.0437) showed statistically significant differences. In the low group, 10 species were more abundant, with *Anaerotaenia torta* (*p* < 0.0001), *Butyricicoccus pullicaecorum* (*p* = 0.0485), and *Oscillibacter valericigenes* (*p* = 0.0054) showing significant differences (Figure 2F and Supplementary Table S5).

To investigate the relationship between motor function and gut microbiota while considering sex differences, we further stratified each motor function group by sex (high group: female *n* = 7, male *n* = 2; medium group: female *n* = 2, male *n* = 8; low group: female *n* = 3, male *n* = 6). In the sex-specific analysis of gut microbiota, alpha diversity showed no significant differences among groups in females. However, in males, evenness factors were significantly increased in the high group for GI samples (Supplementary Figures 1A,B and Supplementary Table S1). At the phylum level, no significant differences were observed in females. In males, the Feces samples showed a distinct difference in Verrucomicrobia abundance, while the GI samples exhibited a significant variation in Firmicutes abundance (Supplementary Figures 1C,D and Supplementary Table S2). The NMDS plot revealed no significant variance differences among motor function groups in both females and males (Supplementary Figures 1E,F and Supplementary Table S3). Overall, these results indicate that significant motor function-related differences in gut microbiota were observed specifically in the male GI samples.

#### 3.2.2 Cognitive function

**Figure 3:**
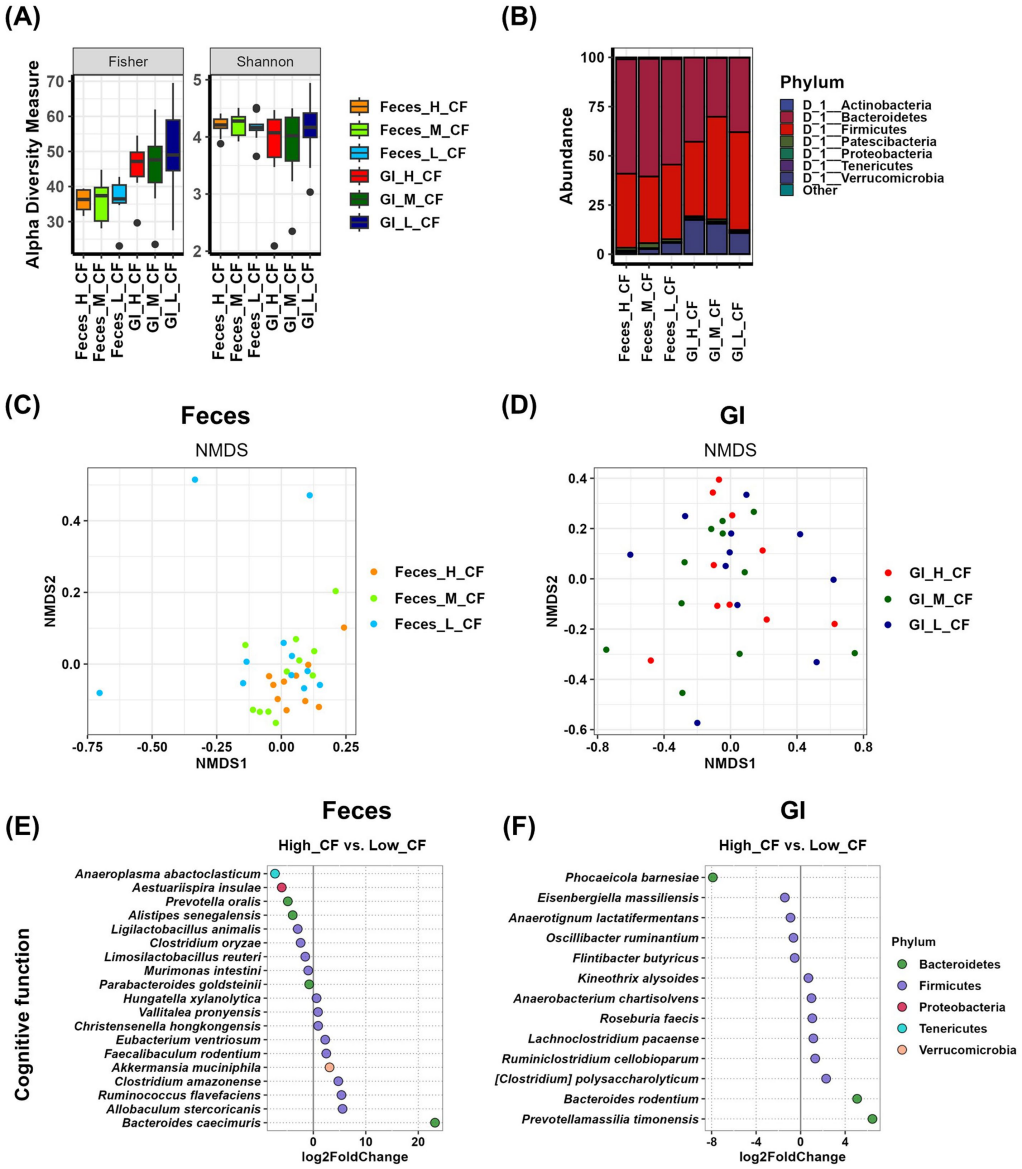
Analysis of the relationship between cognitive function and gut microbiota using Feces and GI tract samples. **(A)** The α-diversity indices of the Feces and GI tract microbiome measured using the Fisher and Shannon method in groups according to cognitive function records are shown in box plots. The mean and *p*-value for each group are presented in Supplementary Table S1. **(B)** Comparison of phylogenetic compositions in groups according to cognitive function records are shown in box plots of the Feces and GI tract microbiome at the phylum level. The mean and *p*-value for each group are presented in Supplementary Table S2. **(C,D)** β-diversity plot of the Feces **(C)** and GI tract **(D)** microbial communities measured using non-metric multidimensional scaling plots in the cognitive function-related group. The results and *p*-values of the PERMANOVA test using Adonis analysis are in Supplementary Table S3. **(E,F)** Log2-fold change in abundance of the most abundant species in the Feces **(E)** and GI tract **(F)** microbial communities of the high and low cognitive function groups as analyzed by DESeq2 differential abundance analysis. Each dot represents a species comparison between the two groups. The results and *p*-values of the DESeq2 test are in Supplementary Table S6. H, high group; M, medium group; L, low group; CF, cognitive function.

To analyze the relationship between gut microbiota and cognitive function in both fecal and gastrointestinal (GI) samples, we conducted an alpha diversity analysis. The results indicated that there were no significant differences in Fisher, Shannon, or other alpha diversity indices among cognitive function groups in either fecal or GI samples (Figure 3A and Supplementary Table S1). At the phylum level, although not statistically significant, the relative abundance of Verrucomicrobia (17.419% in the high group, 15.461% in the medium group, and 10.734% in the low group) in GI samples showed a potential correlation with cognitive function (Figure 3B and Supplementary Table S2). Beta diversity analysis was performed using NMDS plots, and statistical significance was assessed using PERMANOVA tests. The *p*-value for fecal samples was 0.146, while the *p*-value for GI samples was 0.536. Although neither comparison reached statistical significance, fecal samples exhibited more systematic differences between groups than GI samples (Figures 3C,D and Supplementary Table S3). Differential analysis was conducted to identify microbial species enriched in the high and low cognitive function groups. In Feces samples, nine species were enriched in the high group, among which *Anaeroplasma abactoclasticum* (*p* = 0.0016), *Aestuariispira insulae* (*p* = 0.0420), *Ligilactobacillus animalis* (*p* = 0.0000), *Clostridium oryzae* (*p* = 0.0087), and *Murimonas intestini* (*p* = 0.0078) exhibited statistically significant differences. In contrast, 10 species were enriched in the low group, with *Christensenella hongkongensis* (*p* = 0.0124) and *Bacteroides caecimuris* (*p* = 0.0000) showing significant differences (Figure 3E and Supplementary Table S6). In GI samples, five species were more abundant in the high group, with *Phocaeicola barnesiae* (*p* = 0.0050) demonstrating a statistically significant difference. Meanwhile, eight species were enriched in the low group, among which *[Clostridium] polysaccharolyticum* (*p* = 0.0301) and *Prevotellamassilia timonensis* (*p* = 0.0007) exhibited significant differences (Figure 3F and Supplementary Table S6).

To investigate sex differences in the relationship between cognitive function and gut microbiota, we stratified the cognitive function groups by sex (female: high, *n* = 6; medium, *n* = 5; low, *n* = 3; male: high, *n* = 4; medium, *n* = 6; low, *n* = 8). Analysis of alpha diversity revealed no significant differences between cognitive function groups in either females or males (Supplementary Figures 2A,B and Supplementary Table S1). At the phylum level, no significant differences were observed in females. However, in males, microbial composition varied depending on cognitive function levels. In fecal samples, higher cognitive function was associated with an increased abundance of Bacteroidetes. In GI samples, higher cognitive function was linked to a lower abundance of Firmicutes and a significantly higher abundance of Proteobacteria (Supplementary Figures 2C,D and Supplementary Table S2). NMDS analysis showed no significant differences in microbial composition dispersion among cognitive function groups in both females and males. However, a comparison of *p*-values indicated that both females and males exhibited greater group differences in fecal samples than in GI samples (Supplementary Figures 2E,F and Supplementary Table S3).

#### 3.2.3 Emotional function

**Figure 4:**
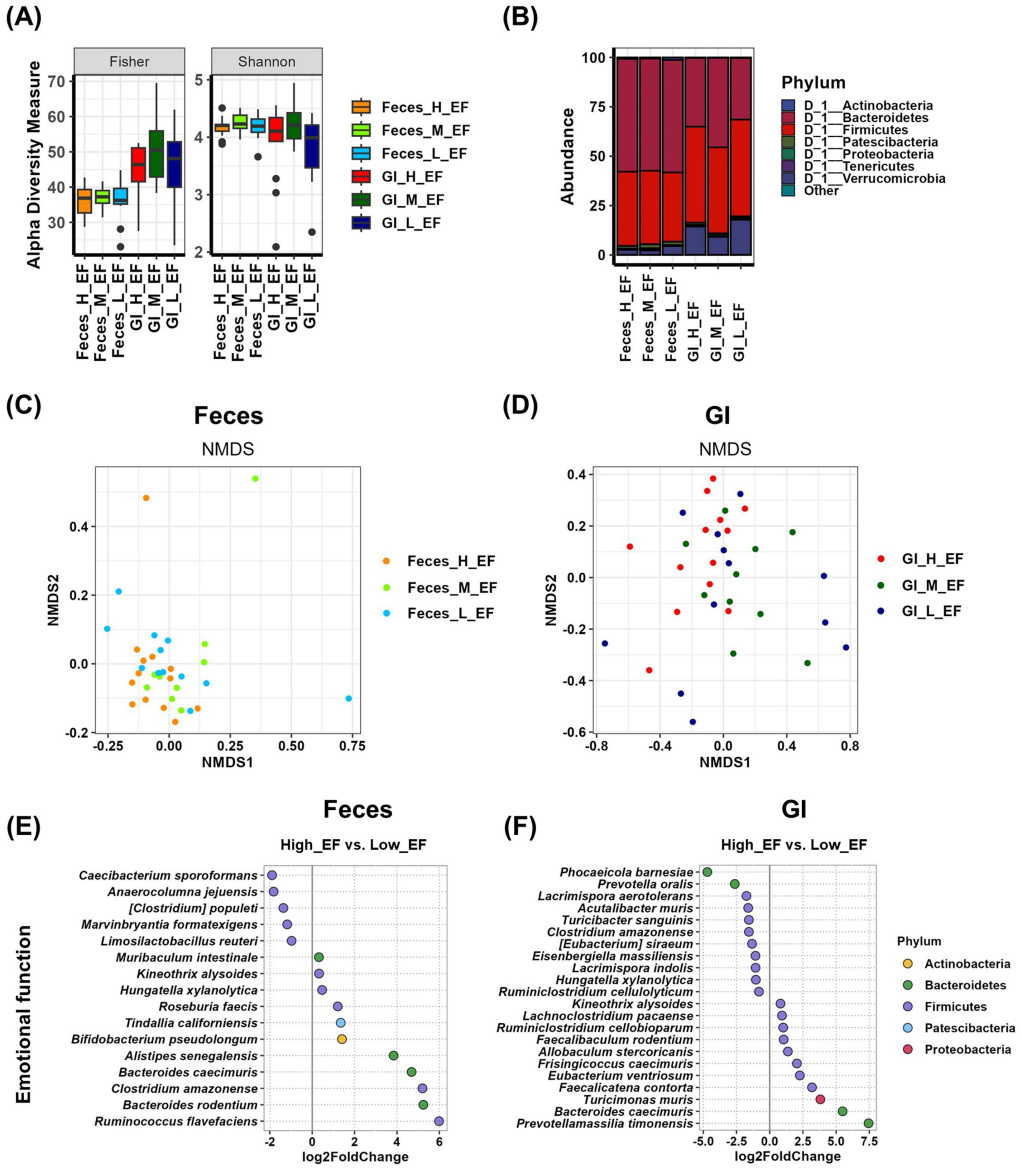
Analysis of the relationship between emotional function and gut microbiota using Feces and GI tract samples. **(A)** The α-diversity indices of the Feces and GI tract microbiome measured using the Fisher and Shannon method in groups according to emotional function records are shown in box plots. The mean and *p*-value for each group are presented in Supplementary Table S1. **(B)** Comparison of phylogenetic compositions in groups according to emotional function records are shown in box plots of the Feces and GI tract microbiome at the phylum level. The mean and *p*-value for each group are presented in Supplementary Table S2. **(C,D)** β-diversity plot of the Feces **(C)** and GI tract **(D)** microbial communities measured using non-metric multidimensional scaling plots in the emotional function-related group. The results and *p*-values of the PERMANOVA test using Adonis analysis are in Supplementary Table S3. **(E,F)** Log2-fold change in abundance of the most abundant species in the Feces **(E)** and GI tract **(F)** microbial communities of the high and low cognitive function groups as analyzed by DESeq2 differential abundance analysis. Each dot represents a species comparison between the two groups. The results and *p*-values of the DESeq2 test are in Supplementary Table S7. H, high group; M, medium group; L, low group; EF, emotional function.

We analyzed the relationship between gut microbial composition in Feces and gastrointestinal (GI) samples and emotional function. Alpha diversity analysis revealed that in GI samples, Simpson, InvSimpson, and Evenness factors were significantly increased in the medium group compared to the high group (*p* < 0.05). However, no other alpha diversity indices showed significant differences among emotional function groups in either Feces or GI samples (Figure 4A and Supplementary Table S1). At the phylum level, Tenericutes were more abundant in the medium group in Feces samples, while in GI samples, Proteobacteria showed a decreased relative abundance in the high group (Figure 4B and Supplementary Table S2). Beta diversity analysis using NMDS plots and PERMANOVA tests showed that the *p*-value for Feces samples was 0.063, whereas the *p*-value for GI samples was 0.025. These results suggest that while Feces samples did not exhibit statistically significant differences, GI samples displayed systematic differences in microbial composition across emotional function groups (Figures 4C,D and Supplementary Table S3). Differential analysis comparing the high and low groups revealed that in Feces samples, five species were enriched in the high group, with *Caecibacterium sporoformans* (*p* = 0.0172), *Anaerocolumna jejuensis* (*p* = 0.0316), *[Clostridium] populeti* (*p* = 0.0399), and *Marvinbryantia formatexigens* (*p* = 0.0166) showing statistically significant differences. In contrast, 11 species were enriched in the low group, among which *Roseburia faecis* (*p* = 0.0167), *Clostridium amazonense* (*p* = 0.0220), and *Ruminococcus flavefaciens* (*p* = 0.0421) exhibited significant differences (Figure 4E and Supplementary Table S7). In GI samples, 11 species were enriched in the high group, with *Turicibacter sanguinis* (*p* = 0.0258) demonstrating a statistically significant difference. Similarly, 11 species were enriched in the low group, among which *Faecalicatena contorta* (*p* = 0.0284) and *Prevotellamassilia timonensis* (*p* = 0.0011) showed significant differences (Figure 4F and Supplementary Table S7).

The gut microbiota analysis of emotional function groups by sex showed no significant differences in alpha diversity in females. In males, only the InvSimpson index in GI samples exhibited a significant difference between the high and low groups (Supplementary Figures 3A,B and Supplementary Table S1). At the phylum level, no significant differences in microbial abundance were observed in either females or males (Supplementary Figures 3C,D and Supplementary Table S2). NMDS analysis revealed minimal group differences in females, while in males, although not statistically significant, fecal samples showed greater group differences than GI samples (Supplementary Figures 3E,F and Supplementary Table S3).

### 3.3 Selection of beneficial gut microorganisms through each behavioral analysis evaluation

We previously identified gut microbiota that were abundant in groups with enhanced and reduced functionality through gut microbiome analysis related to behavioral assessments. Since gut microbiota influence host phenotypes, the differences in the abundance of these specific microbes may have impacted motor function, cognitive function, and emotional function. Based on this, we hypothesized that microbial species enriched in groups with higher motor, cognitive, and emotional function levels may be beneficial bacteria (Table 1).

**Table 1:** Abundance (%) of beneficial candidate microorganisms in each behavioral test.

Test	Sample	Species	Abundance (%)			*p*-value		
			High	Medium	Low	High vs. medium	Medium vs. low	High vs. low
Motor function	Feces	*Aestuariispira insulae*	0.19 ± 0.16	0.01 ± 0.01	0	ns	ns	ns
		*Parabacteroides goldsteinii*	2.46 ± 0.55	0.64 ± 0.23	0.7 ± 0.22	*p* < 0.01	ns	*p* < 0.01
		*Ligilactobacillus animalis*	1.62 ± 0.77	0.87 ± 0.32	0.68 ± 0.15	ns	ns	ns
		*Anaerocolumna jejuensis*	0.49 ± 0.18	0.32 ± 0.15	0.2 ± 0.07	ns	ns	ns
		*Caecibacterium sporoformans*	0.76 ± 0.27	0.49 ± 0.22	0.3 ± 0.11	ns	ns	ns
		*Alistipes finegoldii*	0.97 ± 0.17	0.53 ± 0.08	0.53 ± 0.08	*p* < 0.05	ns	*p* < 0.05
	GI	*Anaerostipes butyraticus*	0.14 ± 0.11	0.06 ± 0.03	0.01 ± 0.01	ns	ns	ns
		*Cellulosilyticum ruminicola*	0.53 ± 0.29	0.11 ± 0.05	0.07 ± 0.03	ns	ns	ns
		*Caecibacterium sporoformans*	0.41 ± 0.19	0.07 ± 0.03	0.06 ± 0.03	ns	ns	ns
		*Mogibacterium neglectum*	0.44 ± 0.18	0.1 ± 0.04	0.09 ± 0.04	ns	ns	ns
		*Faecalicatena contorta*	0.67 ± 0.48	0.16 ± 0.08	0.13 ± 0.11	ns	ns	ns
		*[Clostridium] polysaccharolyticum*	0.4 ± 0.19	0.39 ± 0.26	0.15 ± 0.04	ns	ns	ns
		*Roseburia faecis*	0.87 ± 0.53	0.26 ± 0.07	0.38 ± 0.15	ns	ns	ns
		*Pseudoflavonifractor phocaeensis*	2.17 ± 0.38	1.43 ± 0.23	1.4 ± 0.24	ns	ns	ns
		*Vallitalea pronyensis*	2.2 ± 0.43	3.01 ± 1.52	1.21 ± 0.24	ns	ns	ns
Cognitive function	Feces	*Anaeroplasma abactoclasticum*	0.32 ± 0.13	0.01 ± 0.01	0	*p* < 0.05	ns	*p* < 0.05
		*Aestuariispira insulae*	0.18 ± 0.14	0	0	ns	ns	ns
		*Prevotella oralis*	0.41 ± 0.36	0.24 ± 0.18	0	ns	ns	ns
		*Alistipes senegalensis*	0.45 ± 0.41	0.35 ± 0.35	0	ns	ns	ns
		*Ligilactobacillus animalis*	2.14 ± 0.65	0.82 ± 0.2	0.26 ± 0.08	ns	*p* < 0.05	*p* < 0.01
		*Clostridium oryzae*	0.53 ± 0.12	0.32 ± 0.1	0.1 ± 0.04	ns	*p* < 0.05	*p* < 0.01
		*Limosilactobacillus reuteri*	0.49 ± 0.19	0.24 ± 0.07	0.24 ± 0.14	ns	ns	ns
		*Murimonas intestini*	0.77 ± 0.13	0.71 ± 0.09	0.4 ± 0.08	ns	*p* < 0.05	*p* < 0.05
		*Parabacteroides goldsteinii*	1.45 ± 0.52	1.25 ± 0.44	0.76 ± 0.21	ns	ns	ns
	GI	*Phocaeicola barnesiae*	0.3 ± 0.22	0.41 ± 0.31	0	ns	ns	ns
		*Eisenbergiella massiliensis*	0.4 ± 0.2	0.18 ± 0.05	0.14 ± 0.04	ns	ns	ns
		*Anaerotignum lactatifermentans*	0.42 ± 0.1	0.22 ± 0.06	0.27 ± 0.06	ns	ns	ns
		*Oscillibacter ruminantium*	0.96 ± 0.29	0.65 ± 0.22	0.68 ± 0.16	ns	ns	ns
		*Flintibacter butyricus*	1.16 ± 0.2	0.83 ± 0.17	0.91 ± 0.18	ns	ns	ns
Emotional function	Feces	*Caecibacterium sporoformans*	0.55 ± 0.18	0.72 ± 0.25	0.16 ± 0.08	ns	*p* < 0.05	ns
		*Anaerocolumna jejuensis*	0.36 ± 0.12	0.47 ± 0.17	0.11 ± 0.06	ns	*p* < 0.05	ns
		*[Clostridium] populeti*	0.22 ± 0.09	0.12 ± 0.03	0.08 ± 0.02	ns	ns	ns
		*Marvinbryantia formatexigens*	2.32 ± 0.78	1.68 ± 0.32	1.1 ± 0.35	ns	ns	ns
		*Limosilactobacillus reuteri*	0.52 ± 0.15	0.07 ± 0.02	0.26 ± 0.11	*p* < 0.05	ns	ns
	GI	*Phocaeicola barnesiae*	0.32 ± 0.26	0.51 ± 0.26	0	ns	*p* < 0.05	ns
		*Prevotella oralis*	0.9 ± 0.48	0.08 ± 0.05	0.26 ± 0.15	ns	ns	ns
		*Lacrimispora aerotolerans*	0.83 ± 0.34	0.12 ± 0.06	0.2 ± 0.09	ns	ns	ns
		*Acutalibacter muris*	0.26 ± 0.09	0.08 ± 0.03	0.42 ± 0.36	ns	ns	ns
		*Turicibacter sanguinis*	2.63 ± 1.09	0.91 ± 0.28	0.69 ± 0.2	ns	ns	ns
		*Clostridium amazonense*	1.07 ± 0.37	1.01 ± 0.22	0.46 ± 0.26	ns	ns	ns
		*[Eubacterium] siraeum*	0.93 ± 0.25	0.41 ± 0.11	0.33 ± 0.09	ns	ns	*p* < 0.05
		*Eisenbergiella massiliensis*	0.34 ± 0.15	0.13 ± 0.06	0.15 ± 0.05	ns	ns	ns
		*Lacrimispora indolis*	2.58 ± 1.03	2.13 ± 1.11	1.14 ± 0.37	ns	ns	ns
		*Hungatella xylanolytica*	3.63 ± 1.24	3.18 ± 1.38	1.58 ± 0.71	ns	ns	ns
		*Ruminiclostridium cellulolyticum*	0.77 ± 0.2	0.78 ± 0.26	0.4 ± 0.11	ns	ns	ns

ns, not significant.

Analysis of Feces samples related to motor function revealed that *Aestuariispira insulae*, *Parabacteroides goldsteinii*, *Ligilactobacillus animalis*, *Anaerocolumna jejuensis*, *Caecibacterium sporoformans*, and *Alistipes finegoldii* were more abundant in the high group (Figure 2E and Table 1). Among these, *P. goldsteinii* (high vs. low, *p* < 0.01) and *A. finegoldii* (high vs. low, *p* < 0.05) showed significant differences in % abundance across groups, suggesting their potential role as beneficial bacteria for motor function. In contrast, the analysis using GI samples identified *Anaerostipes butyraticus*, *Cellulosilyticum ruminicola*, *Caecibacterium sporoformans*, *Mogibacterium neglectum*, *Faecalicatena contorta*, *[Clostridium] polysaccharolyticum*, *Roseburia faecis*, *Pseudoflavonifractor phocaeensis*, and *Vallitalea pronyensis* as more abundant in the high group (Figure 2F and Table 1), but their % abundance did not show statistical significance.

In terms of cognitive function, the analysis of Feces samples showed that *Anaeroplasma abactoclasticum*, *Aestuariispira insulae*, *Prevotella oralis*, *Alistipes senegalensis*, *Ligilactobacillus animalis*, *Clostridium oryzae*, *Limosilactobacillus reuteri*, *Murimonas intestini*, and *Parabacteroides goldsteinii* were enriched in the high group (Figure 3E and Table 1). Among these, *A. abactoclasticum* (high vs. low, *p* < 0.05), *L. animalis* (high vs. low, *p* < 0.01), *C. oryzae* (high vs. low, *p* < 0.01), and *M. intestine* (high vs. low, *p* < 0.05) exhibited significant differences in % abundance, indicating their potential association with cognitive function. In GI samples, *Phocaeicola barnesiae*, *Eisenbergiella massiliensis*, *Anaerotignum lactatifermentans*, *Oscillibacter ruminantium*, and *Flintibacter butyricus* were more abundant in the high group (Figure 3F and Table 1). However, no statistically significant differences were observed in % abundance.

In case of emotional function, Feces sample analysis revealed that *Caecibacterium sporoformans*, *Anaerocolumna jejuensis*, *[Clostridium] populeti*, *Marvinbryantia formatexigens*, and *Limosilactobacillus reuteri* were more abundant in the high group (Figure 4E and Table 1), though there were no significant differences in % abundance between the high and low groups. In GI samples, *Phocaeicola barnesiae*, *Prevotella oralis*, *Lacrimispora aerotolerans*, *Acutalibacter muris*, *Turicibacter sanguinis*, *Clostridium amazonense*, *[Eubacterium] siraeum*, *Eisenbergiella massiliensis*, *Lacrimispora indolis*, *Hungatella xylanolytica*, and *Ruminiclostridium cellulolyticum* were found to be enriched in the high group (Figure 4F and Table 1). Among these, *[Eubacterium] siraeum* (high vs. low, *p* < 0.05) exhibited statistical significance, suggesting a potential beneficial role in emotional function.

To further assess the potential impact of these selected beneficial microbes on host phenotypes, we quantified microbiability (He et al., 2022), which estimates the proportion of phenotypic variance explained by microbial abundance (Table 2). The microbiability analysis demonstrated that microbes identified from Feces and GI samples exhibited different levels of influence on motor, cognitive, and emotional functions. In particular, the microbial diversity of the microorganisms selected from the Feces sample was higher than the GI sample, and the microbial diversity of the microorganisms selected from the GI sample was higher than that from the Feces sample, indicating that the choice of sampling method can influence the identification of microbes that are more strongly associated with host phenotypic traits (Table 2). Overall, our findings highlight that the selection of beneficial gut microbes and their estimated contribution to host phenotypic variance are dependent on the sampling method. These results emphasize the importance of considering sample type when studying gut microbiota’s functional role in host physiology.

**Table 2:** Microbiavailability of microorganisms expected to be beneficial for motor, cognitive, and emotional functions.

Test	Sample	Species	Behavior test	Feces		GI	
				Microbiability	*p*-value	Microbiability	*p*-value
Motor function	Feces	*Aestuariispira insulae*	Rotarod	0.1580	0.0362	0.3029	0.0024
			Wire suspension test	0.2137	0.0133	0.0011	0.8687
		*Parabacteroides goldsteinii*	Rotarod	0.3432	0.0011	0.0404	0.3050
			Wire suspension test	0.4886	0.0000	0.0196	0.4779
		*Ligilactobacillus animalis*	Rotarod	0.0088	0.6353	0.0383	0.3183
			Wire suspension test	0.0097	0.6180	0.0954	0.1098
		*Anaerocolumna jejuensis*	Rotarod	0.2578	0.0058	0.0146	0.5406
			Wire suspension test	0.0874	0.1266	0.0079	0.6521
		*Caecibacterium sporoformans*	Rotarod	0.2552	0.0061	0.0150	0.5341
			Wire suspension test	0.1047	0.0931	0.0458	0.2740
		*Alistipes finegoldii*	Rotarod	0.2286	0.0101	0.0215	0.4570
			Wire suspension test	0.4874	0.0000	0.0014	0.8516
	GI	*Anaerostipes butyraticus*	Rotarod	0.1006	0.1000	0.0010	0.8715
			Wire suspension test	0.0355	0.3373	0.0071	0.6705
		*Cellulosilyticum ruminicola*	Rotarod	0.0016	0.8376	0.2454	0.0074
			Wire suspension test	0.0006	0.9013	0.1591	0.0355
		*Caecibacterium sporoformans*	Rotarod	0.2552	0.0061	0.0150	0.5341
			Wire suspension test	0.1047	0.0931	0.0458	0.2740
		*Mogibacterium neglectum*	Rotarod	0.0183	0.4928	0.2684	0.0047
			Wire suspension test	0.0016	0.8391	0.1652	0.0319
		*Faecalicatena contorta*	Rotarod	—	—	0.0001	0.9653
			Wire suspension test	—	—	0.0955	0.1095
		*[Clostridium] polysaccharolyticum*	Rotarod	0.0437	0.2856	0.0106	0.6016
			Wire suspension test	0.0014	0.8517	0.0009	0.8770
		*Roseburia faecis*	Rotarod	0.0396	0.3103	0.0001	0.9535
			Wire suspension test	0.0015	0.8474	0.0019	0.8244
		*Pseudoflavonifractor phocaeensis*	Rotarod	0.0216	0.4560	0.1384	0.0512
			Wire suspension test	0.0001	0.9652	0.0000	0.9792
		*Vallitalea pronyensis*	Rotarod	0.0511	0.2472	0.0220	0.4516
			Wire suspension test	0.0189	0.4857	0.0056	0.7051
Cognitive function	Feces	*Anaeroplasma abactoclasticum*	Y-maze	0.1630	0.0219	0.0173	0.4726
		*Aestuariispira insulae*	Y-maze	0.0733	0.1338	0.0128	0.5370
		*Prevotella oralis*	Y-maze	0.0277	0.3623	0.0056	0.6830
		*Alistipes senegalensis*	Y-maze	0.0022	0.7987	0.0004	0.9138
		*Ligilactobacillus animalis*	Y-maze	0.2863	0.0016	0.0063	0.6669
		*Clostridium oryzae*	Y-maze	0.1124	0.0607	0.0046	0.7120
		*Limosilactobacillus reuteri*	Y-maze	0.0761	0.1265	0.0181	0.4630
		*Murimonas intestini*	Y-maze	0.0889	0.0973	0.0139	0.5207
		*Parabacteroides goldsteinii*	Y-maze	0.0134	0.5274	0.0188	0.4541
	GI	*Phocaeicola barnesiae*	Y-maze	0.0010	0.8639	0.0289	0.3525
		*Eisenbergiella massiliensis*	Y-maze	0.0029	0.7698	0.0205	0.4347
		*Anaerotignum lactatifermentans*	Y-maze	0.0165	0.4837	0.1539	0.0264
		*Oscillibacter ruminantium*	Y-maze	0.0060	0.6745	0.0015	0.8323
		*Flintibacter butyricus*	Y-maze	0.0001	0.9634	0.0101	0.5839
Emotional function	Feces	*Caecibacterium sporoformans*	Tail suspension test	0.1114	0.0500	0.0051	0.6846
		*Anaerocolumna jejuensis*	Tail suspension test	0.0919	0.0767	0.0000	0.9833
		*[Clostridium] populeti*	Tail suspension test	0.0723	0.1184	0.0103	0.5611
		*Marvinbryantia formatexigens*	Tail suspension test	0.0402	0.2484	0.0207	0.4099
		*Limosilactobacillus reuteri*	Tail suspension test	0.0882	0.0833	0.0100	0.5677
	GI	*Phocaeicola barnesiae*	Tail suspension test	0.0233	0.3817	0.0110	0.5494
		*Prevotella oralis*	Tail suspension test	0.1155	0.0458	0.0829	0.0934
		*Lacrimispora aerotolerans*	Tail suspension test	0.0224	0.3910	0.1057	0.0567
		*Acutalibacter muris*	Tail suspension test	0.0226	0.3886	0.0073	0.6263
		*Turicibacter sanguinis*	Tail suspension test	0.0015	0.8252	0.0508	0.1930
		*Clostridium amazonense*	Tail suspension test	0.0276	0.3405	0.0824	0.0945
		*[Eubacterium] siraeum*	Tail suspension test	0.0175	0.4487	0.1873	0.0094
		*Eisenbergiella massiliensis*	Tail suspension test	0.0477	0.2076	0.0982	0.0668
		*Lacrimispora indolis*	Tail suspension test	0.0636	0.1438	0.0546	0.1766
		*Hungatella xylanolytica*	Tail suspension test	0.0634	0.1445	0.0201	0.4167
		*Ruminiclostridium cellulolyticum*	Tail suspension test	0.0009	0.8670	0.0907	0.0787

## 4 Discussion

Our findings highlight the importance of sample collection methods in microbiome research, particularly when investigating the relationship between the gut microbiome and host phenotype. In our study, gastrointestinal (GI) samples were collected by scraping luminal contents along the mucosal surface from the stomach to the end of the large intestine, ensuring that both luminal and potentially mucosa-associated microbiota were included. However, we acknowledge that our approach does not fully replace direct mucosal microbiota sampling, such as biopsy-based methods. Future studies should incorporate these advanced techniques to further characterize the interactions between resident microbiota and host physiology (David et al., 2014). These differences suggest that a comprehensive gut microbiome analysis may benefit from incorporating both sample types to capture a more complete microbial landscape. GI samples provide insights into resident microbial communities that interact more directly with host tissues, whereas fecal samples capture microbial dynamics and environmental fluctuations in the gut. Integrating both sample types may allow for a more holistic understanding of microbiota-host interactions.

Furthermore, our comparative analysis revealed that microbial composition in GI samples was more reflective of host behavioral phenotypes than fecal samples. Alpha-diversity indices demonstrated significantly greater microbial richness in GI samples, while beta-diversity analyses confirmed distinct microbial clustering between fecal and GI samples. Additionally, taxonomic differences showed a notable increase in Firmicutes and Verrucomicrobia and a decrease in Bacteroidetes in GI samples, suggesting that fecal samples may disproportionately represent transient or shed bacteria rather than the resident microbial community. These findings align with prior research indicating that different gut regions harbor distinct microbial populations, which may contribute uniquely to host physiology (Kim, 2013; Rhee et al., 2009).

Interestingly, blood metabolite analysis revealed significant body weight differences among motor and emotional function groups, with lower body weight observed in high-performing groups (*p* < 0.01 and *p* < 0.001, respectively). Variations in lipid profiles, including total cholesterol and LDL levels, were significant in the emotional function group (*p* < 0.01), suggesting that metabolic factors may also contribute to microbiota-host interactions relevant to behavior. Although these metabolites remained within normal physiological ranges, their potential influence on gut microbiota and behavioral traits warrants further investigation (Zoetendal et al., 2002). These results highlight the necessity of integrating metabolic profiling with microbiome analysis to better understand how systemic factors contribute to behavioral phenotypes.

Differential abundance analyses provided insights into microbial species potentially associated with motor, cognitive, and emotional functions. While several species were enriched in the high group, indicating a possible link to improved function, we emphasize that these findings are correlative. Establishing these species as beneficial microbes requires further validation through microbiota transplantation, metabolomic profiling, and mechanistic studies. Figures 2–4 highlight the microbial candidates associated with motor, cognitive, and emotional functions, comparing fecal and GI samples. In motor function analysis, six species were identified in fecal samples, whereas nine species were identified in GI samples (Figure 2). Interestingly, *Caecibacterium sporoformans* was the only species found in both fecal and GI samples, suggesting that while some microbial species are present across both sample types, their relative abundances and interactions with the host may differ significantly depending on the environment. This highlights the importance of considering both transient and resident microbial populations in understanding host-microbiota interactions. However, the majority of identified species were distinct between fecal and GI samples, reinforcing the notion that GI microbiota may provide a more stable and physiologically relevant representation of microbial influences on host behavior (Duncan et al., 2004; Shobar et al., 2016).

For cognitive function, differential analysis identified nine species enriched in fecal samples but only five species in GI samples (Figure 3). Importantly, no microbial species were shared between these two sample types, further emphasizing the distinct microbiota profiles across different gut regions (Zoetendal et al., 2001). The greater number of differentially abundant species in fecal samples may suggest that transient microbiota contributes dynamically to cognitive processes through various metabolic pathways. However, GI samples provided a more consistent microbial signature related to cognitive function, reinforcing the idea that gastrointestinal microbiota offers a stable foundation for host-microbiota interactions (Cryan et al., 2019). This aligns with previous findings indicating that GI samples more accurately reflect the microbial composition of the gut ecosystem compared to fecal samples (Tang et al., 2020). Studies have shown that fecal microbiota primarily represent transient bacterial populations, whereas GI samples provide insights into microbial communities that are actively interacting with host tissues, immune responses, and metabolic processes. These findings contribute to the growing body of research suggesting that GI-resident microbiota play a crucial role in host physiological and behavioral regulation (Sittipo et al., 2022).

Emotional function analysis demonstrated a similar trend, with five species identified in fecal samples and 11 species identified in GI samples showing increased abundance in the high-performing group (Figure 4). Notably, no common species were observed between fecal and GI samples, suggesting that the microbial communities in these two compartments are functionally distinct. This further supports the idea that GI microbiota may provide a more comprehensive representation of microbial influences on host emotional traits (Chaudhry et al., 2023). Unlike fecal samples, which primarily contain transient bacteria shed from the colon, GI samples reflect resident microbial populations that are more likely to engage in sustained interactions with the host’s immune system and nervous system (Chen et al., 2021; Xiong et al., 2023). Studies have shown that mucosa-associated microbiota play a crucial role in neurotransmitter production, stress response regulation, and immune signaling, all of which are critical factors in emotional behavior (Loh et al., 2024). Therefore, the lack of overlapping species highlights the unique role of GI microbiota in modulating emotional function, reinforcing the importance of sampling from the gut environment rather than relying solely on fecal microbiota. The greater number of differentially abundant species in GI samples strengthens the hypothesis that GI microbiota is more directly involved in regulating host behavioral phenotypes, potentially through their interactions with the gut-brain axis.

While our study provides valuable insights into the relationship between gut microbiota and behavioral functions, it is not without limitations. First, although our GI samples included luminal and potentially mucosa-associated microbiota, we did not perform direct mucosal microbiota sampling, such as epithelial cell isolation or biopsy-based methods. This limits our ability to fully characterize mucosa-associated microbial communities, which are known to interact closely with host immune and nervous systems. Future studies incorporating direct mucosal sampling will be essential to further elucidate these interactions. Second, our analysis was conducted on a limited sample size within a controlled environment, which may not fully capture the complexity of microbiota-host interactions under diverse conditions. Third, while we identified microbial candidates associated with motor, cognitive, and emotional traits, functional validation of their roles requires further mechanistic studies, such as microbiota transplantation, metabolomic profiling, or host-microbiota interaction assays. Additionally, longitudinal studies assessing temporal changes in microbiota composition could provide a deeper understanding of microbiota dynamics in behavioral regulation.

By providing a comparative analysis of fecal and GI microbiota in relation to behavioral traits, our study contributes to the growing understanding of microbiota-gut-brain interactions. These findings suggest that gut microbiome-based therapeutic strategies should consider both transient and resident microbial populations to optimize interventions for behavioral and neurological disorders. Future research should further investigate how microbiome-targeted interventions, such as dietary modifications, probiotic treatments, and microbiota transplantation, may differentially influence host behavior depending on microbial localization within the gut. These findings have potential implications for the development of microbiota-based therapeutic strategies aimed at improving cognitive and emotional health.

These findings underscore the need for careful consideration of sample collection methods in microbiome research. The differential microbial composition observed between fecal and GI samples suggests that gastrointestinal samples may provide distinct but complementary insights into host phenotypes. Given the dynamic microbial ecosystem along the GI tract, relying solely on fecal microbiota may lead to incomplete conclusions regarding microbial contributions to physiology and behavior. Moving forward, incorporating both fecal and GI samples into microbiome research may provide a more comprehensive understanding of the gut microbiome’s role in regulating host health. While GI samples may capture more stable microbial populations, fecal samples also provide critical insights into microbial dynamics and gut environment changes. Future studies should consider analyzing both sample types to fully assess gut microbiota-host interactions.

## 5 Conclusion

Our study highlights the importance of sample collection methods in microbiome research and their influence on the interpretation of gut microbiota-host interactions. By comparing microbiota composition between fecal and gastrointestinal (GI) samples, we demonstrated that GI samples more accurately reflect resident microbial communities, whereas fecal samples primarily capture transient bacterial populations. Furthermore, GI microbiota exhibited a stronger association with host motor, cognitive, and emotional functions, suggesting their potential role in regulating behavioral phenotypes. However, as our findings are correlative, further studies incorporating microbiota transplantation, metabolomic profiling, and mechanistic analyses are needed to validate the functional significance of these microbial candidates. Additionally, the choice between fecal and GI sampling should be guided by research objectives, as each provides distinct insights into gut microbiota composition and function. Integrating both sample types allows for a more comprehensive understanding of host-microbiota interactions and may enhance the accuracy of microbiome-based therapeutic strategies. Moving forward, microbiome-targeted interventions, such as dietary modifications, probiotics, and microbiota transplantation, should consider the localization of microbial populations to optimize therapeutic strategies for improving behavioral and neurological health.

## Data Availability

Raw data from blood serum analyses of mice, the raw data for mouse behavior tests, and the 16S rRNA sequencing taxonomy profile data were deposited at figshare (https://doi.org/10.6084/m9.figshare.25744062.v1). The 16S rRNA sequencing raw data have been deposited in NCBI’s SRA database accession number PRJNA1104424 (https://identifiers.org/bioproject:PRJNA1104424).

## Footnotes

^1^ https://www.arb-silva.de/
