# Supplementary material for: Comparison of metagenomic analysis of fecal and gastrointestinal tract samples for identifying beneficial gut microorganisms
Source: Front Microbiol. 2025 Mar 26;16:1533580. doi: 10.3389/fmicb.2025.1533580 (PMC11979174; doi:10.3389/fmicb.2025.1533580)
Supplement: Supplementary file 2 [file Data_Sheet_1.docx]

**Supplementary Information**

**Comparison of metagenomic analysis of fecal and gastrointestinal tract samples for identifying beneficial gut microorganisms**

Ji-Seon Ahn, Eui-Jeong Han, Hea-Jong Chung^*^

**Figure S1**

**
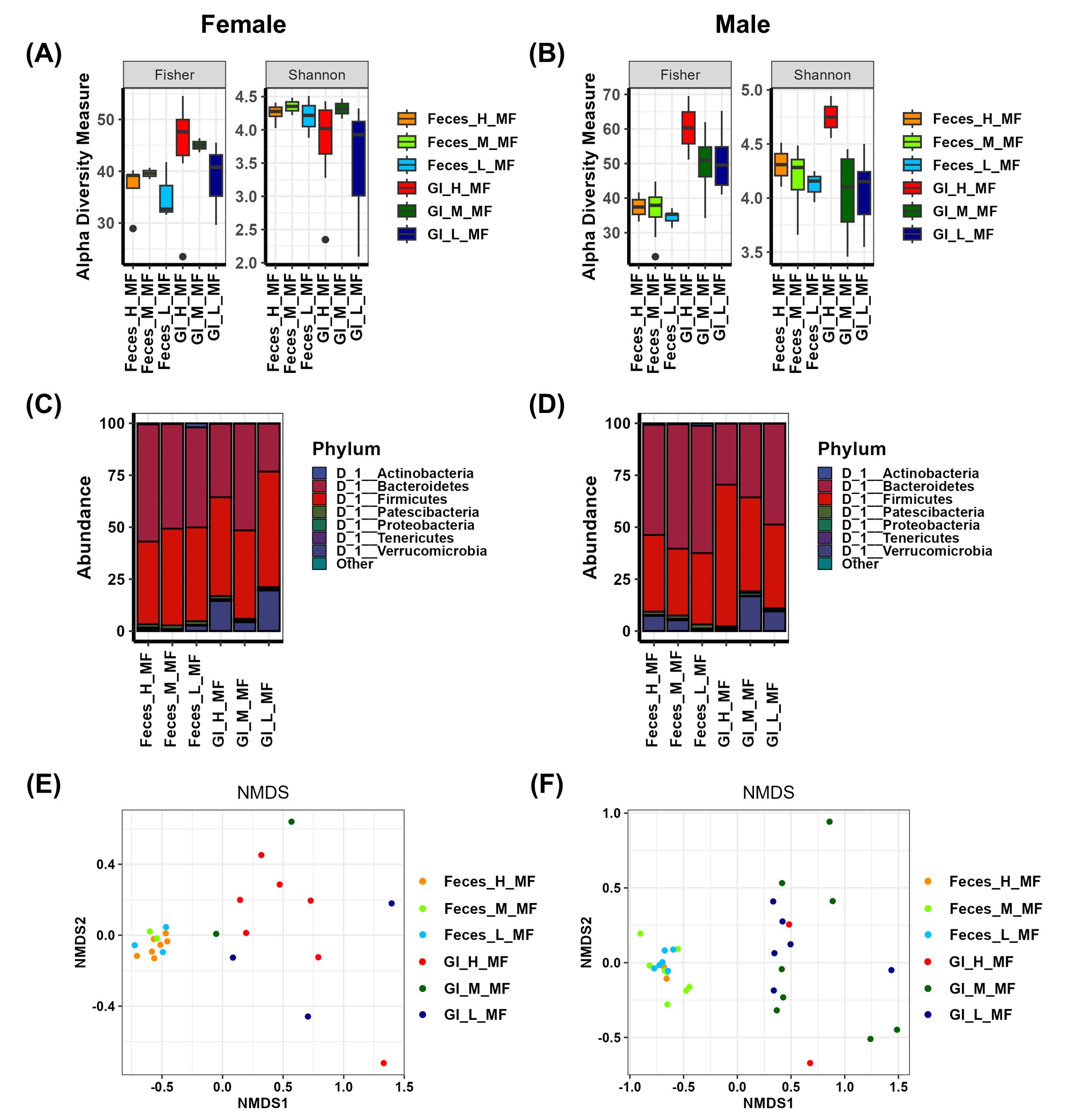
**

**Supplementary Figure 1. Analysis of the relationship between motor function and gut microbiota using Feces and GI tract samples.** (**A,B**) The α-diversity indices of the Feces and GI tract microbiome measured using the Fisher and Shannon method in groups according to motor function records are shown in box plots. α-diversities in females (**A**) and males (**B**) are shown, respectively. The mean and *p*-value for each group are presented in supplementary Table S1. (**C,D**) Comparison of phylogenetic compositions in groups according to motor function records are shown in box plots of the Feces and GI tract microbiome at the phylum level. % Abundance of each phylum in females (**C**) and males (**D**) are shown, respectively. The mean and *p*-value for each group are presented in supplementary Table S2. (**E,F**) β-diversity plot of the Feces and GI tract microbial communities measured using non-metric multidimensional scaling plots in the motor function-related group. NMDS plots in females (**E**) and males (**F**) are shown, respectively. The results and *p*-values of the PERMANOVA test using Adonis analysis are in Supplementary Table S3. H: high group, M: medium group, L: low group, MF: motor function.

**Figure S2**

**
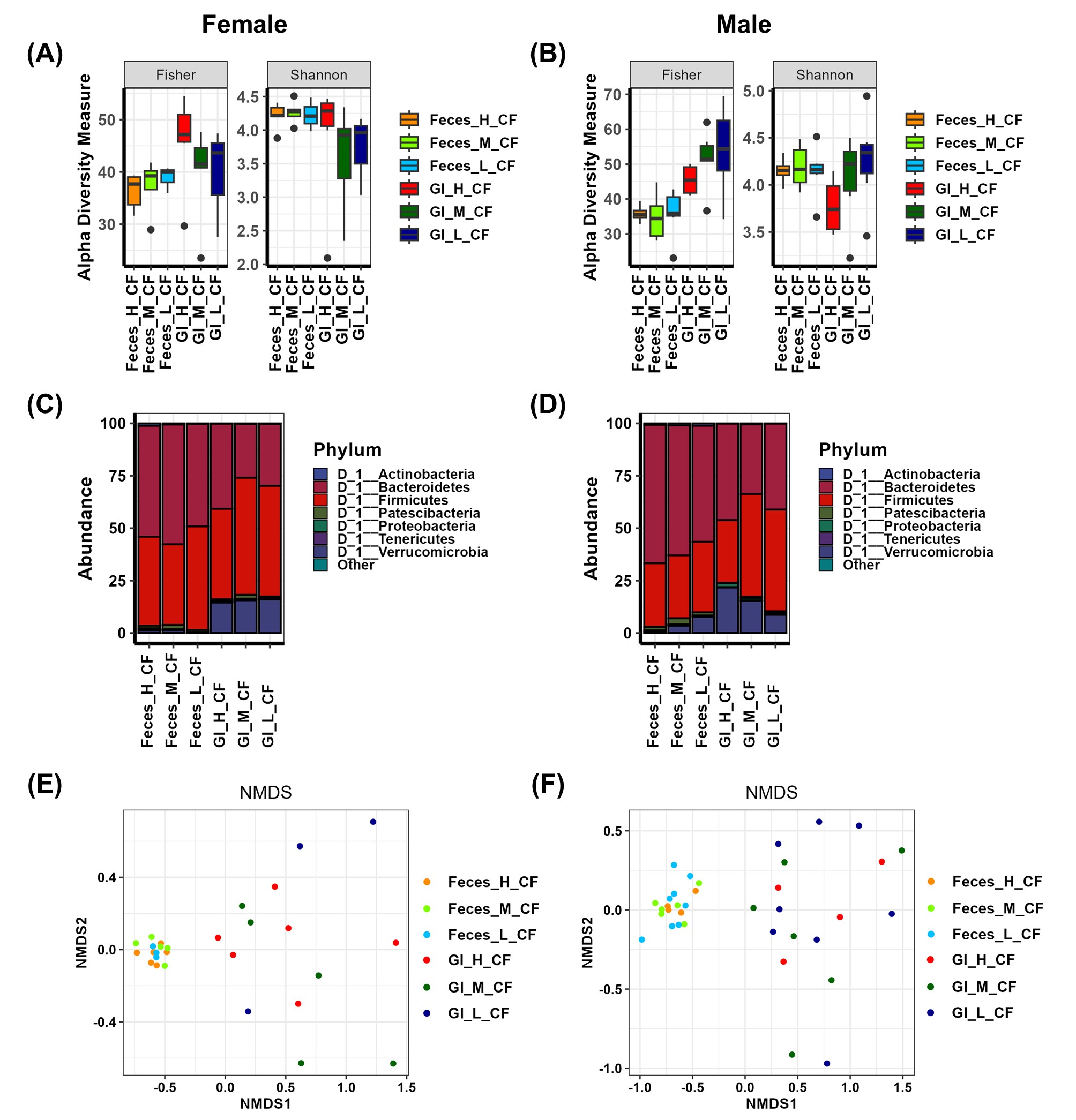
**

**Supplementary Figure 2. Analysis of the relationship between cognitive function and gut microbiota using Feces and GI tract samples.** (**A,B**) The α-diversity indices of the Feces and GI tract microbiome measured using the Fisher and Shannon method in groups according to cognitive function records are shown in box plots. α-diversities in females (**A**) and males (**B**) are shown, respectively. The mean and *p*-value for each group are presented in supplementary Table S1. (**C,D**) Comparison of phylogenetic compositions in groups according to cognitive function records are shown in box plots of the Feces and GI tract microbiome at the phylum level. % Abundance of each phylum in females (**C**) and males (**D**) are shown, respectively. The mean and *p*-value for each group are presented in supplementary Table S2. (**E,F**) β-diversity plot of the Feces and GI tract microbial communities measured using non-metric multidimensional scaling plots in the cognitive function-related group. NMDS plots in females (**E**) and males (**F**) are shown, respectively. The results and *p*-values of the PERMANOVA test using Adonis analysis are in Supplementary Table S3. H: high group, M: medium group, L: low group, CF: cognitive function.

**Figure S3**

**
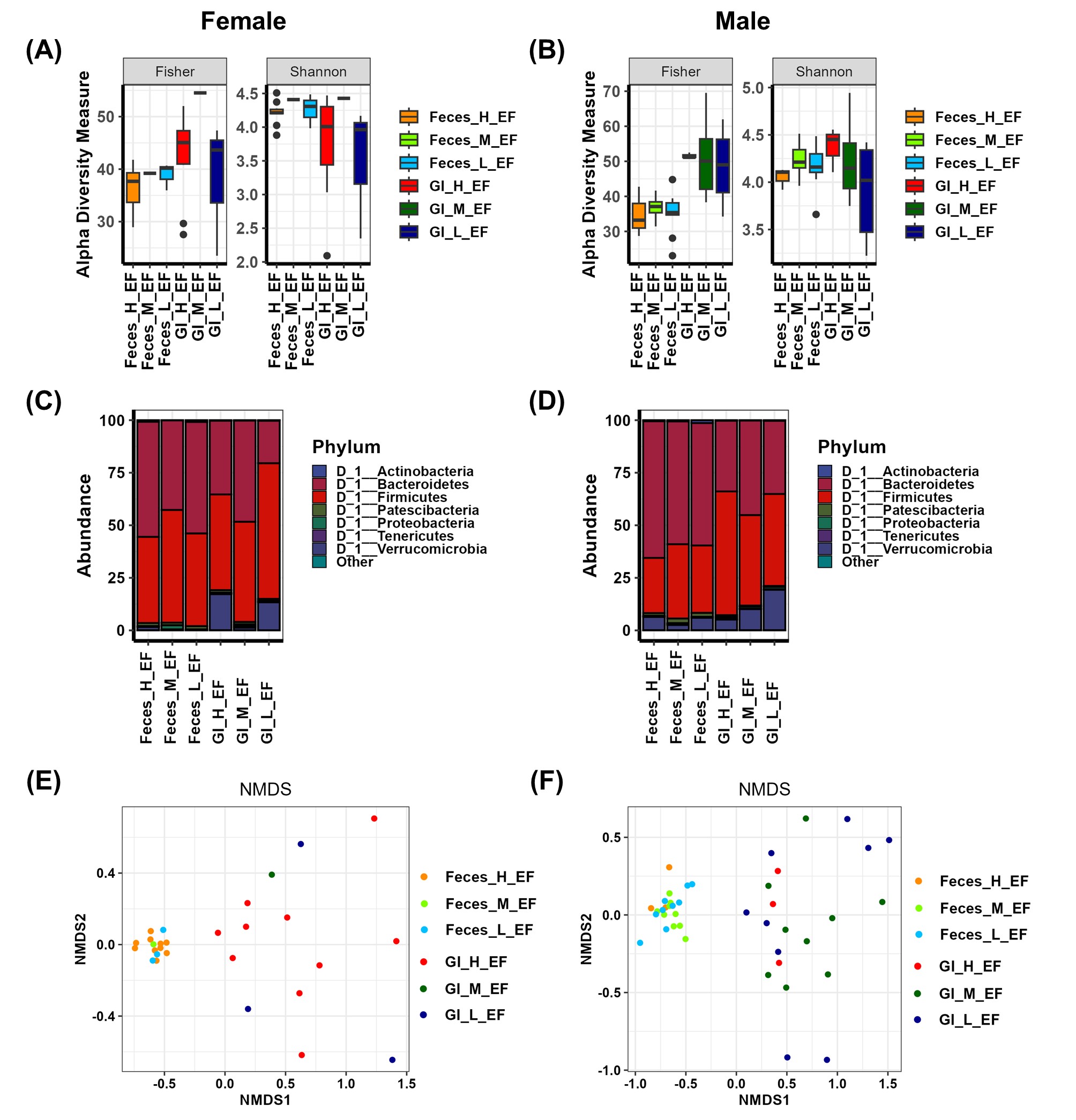
**

**Supplementary Figure 3. Analysis of the relationship between emotional function and gut microbiota using Feces and GI tract samples.** (**A,B**) The α-diversity indices of the Feces and GI tract microbiome measured using the Fisher and Shannon method in groups according to emotional function records are shown in box plots. α-diversities in females (**A**) and males (**B**) are shown, respectively. The mean and *p*-value for each group are presented in supplementary Table S1. (**C,D**) Comparison of phylogenetic compositions in groups according to emotional function records are shown in box plots of the Feces and GI tract microbiome at the phylum level. % Abundance of each phylum in females (**C**) and males (**D**) are shown, respectively. The mean and *p*-value for each group are presented in supplementary Table S2. (**E,F**) β-diversity plot of the Feces and GI tract microbial communities measured using non-metric multidimensional scaling plots in the emotional function-related group. NMDS plots in females (**E**) and males (**F**) are shown, respectively. The results and *p*-values of the PERMANOVA test using Adonis analysis are in Supplementary Table S3. H: high group, M: medium group, L: low group, EF: emotional function.
